# Cannabis-Induced Third-Degree AV Block

**DOI:** 10.1155/2019/5037356

**Published:** 2019-09-16

**Authors:** Jan M. Van Keer

**Affiliations:** ^1^Department of Cardiology, University Hospitals Leuven, Belgium; ^2^Department of Microbiology, Immunology and Transplantation, KU Leuven, University of Leuven, Belgium

## Abstract

**Background:**

Cannabis (marijuana) is the most widely consumed illicit drug in Europe. However, many are unaware of its potential cardiovascular side effects.

**Case Report:**

A 19-year-old man presented to the emergency department with palpitations and presyncope after smoking cannabis. A third-degree atrioventricular block (complete heart block) was diagnosed. We believe cannabis exposure to have been the likely cause. Extensive work-up—including Borrelia and auto-immune serology, CT coronary angiography, magnetic resonance imaging, and electrophysiological study—was negative. The patient was initially treated with IV isoprenaline. Within one day, the bradycardia spontaneously resolved. The patient was advised to quit using cannabis. No further therapy was initiated. We discuss the clinical presentation, pathophysiology, and evidence from the literature linking cannabis exposure to bradycardia.

**Conclusion:**

We describe a case of third-degree atrioventricular block after cannabis use. Emergency physicians should be aware of the potential cardiovascular side effects of this drug.

## 1. Introduction

Third-degree atrioventricular block is defined by a lack of transmission of the impulse from the atria to the ventricles, due to an anatomical or functional impairment in the conduction system. The etiology includes ischemic heart disease, cardiomyopathy, myocarditis, degenerative disease (Lenegre-Lev syndrome), infiltrative processes, congenital heart disease, congenital AV block, metabolic causes (hyperkalemia, hypoxia, thyroid dysfunction), increased vagal tone, cardiac procedures, drugs (beta-blockers, nondihydropyridine calcium channel blockers, antiarrhythmics) and toxins (cardiac glycosides). The incidence increases with advancing age [1]. A few reports have described atrioventricular block in young patients without underlying cardiac disease, following cannabis use [2–6].

Cannabis (marijuana) is the most widely consumed illicit drug in Europe [7]. While the psychoactive effects of cannabis are well known, few people are aware of its potential cardiovascular side effects. Many patients are seeking treatment for their cannabis use disorder, but somatic investigations are seldom performed [8].

We describe a patient presenting to the emergency department with third-degree atrioventricular block (complete heart block) after cannabis use.

## 2. Case Presentation

A 19-year-old man presented to the emergency department with palpitations and presyncope. He experienced three episodes of near complete loss of consciousness in the last few hours prior to presentation. Each episode had lasted for less than thirty seconds and had been accompanied by palpitations. One hour before symptom onset, the patient had smoked a joint. He had been using cannabis since 2 years, on average 2–3 joints per week. Never before had he experienced syncope or presyncope. He had no past medical history except for autism spectrum disorder diagnosed at age 11 and he was not taking any medication. Family history was negative for sudden death or pacemaker implantation.

On clinical examination, the patient appeared well. He had no fever (36.9°C), oxygen saturation at room air was 99%, respiratory rate 20/min, and blood pressure 120/70 mmHg. On initial examination, heart rate was 100 beats per minute. Heart sounds were normal without murmurs, lung auscultation was clear and the remainder of the clinical exam was unremarkable.

**Figure 1:**
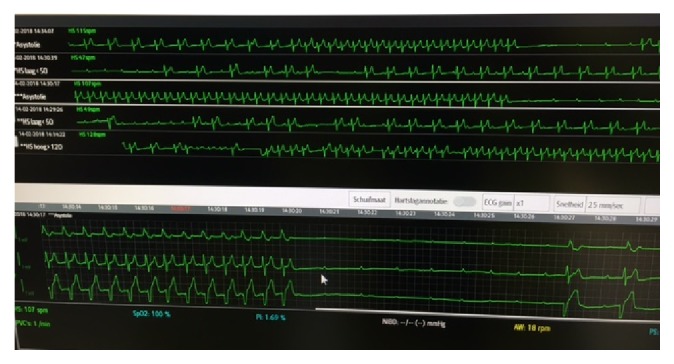
Third-degree atrioventricular block. During observation at the emergency department, multiple episodes of third-degree atrioventricular block were noted, the longest of which lasted 7.2 s (arrow).

Initial electrocardiogram (ECG) showed intermittent sinus rhythm and junctional tachycardia up to 120 beats per minute. During observation, ECG monitoring showed several episodes of third-degree atrioventricular block, with underlying sinus rhythm but without ventricular escape. These episodes, the longest of which lasted 7.2 s (Figure 1) provoked the same symptoms as the patient had described.

Urinary 11-nor-9-carboxy-Δ^9^-tetrahydrocannabinol (THC-COOH) was 110 ng/mL (Cannabinoids II SemiQuantitative 50® Assay, run on COBAS INTEGRA® 800 Analyzer, Roche Diagnostics). Urine toxicology screen was negative for cocaine, amphetamines, opiates, neuroleptics, or antidepressants. Blood digoxin level was negative. High sensitivity troponin was 0.018 mcg/L (reference value, <0.013), with a rise to 0.027 mcg/L three hours later and gradual decline thereafter. NT-proBNP was 176 ng/L (reference value, <115). C-reactive protein was normal (0.7 mg/L, reference value, <5.0). Hemoglobin (15.6 g/dL, reference value, 14.0–18.0), white blood cell count (7.24 ^*∗*^ 10^9^/L, reference value, 4.00–10.00), and platelets (235 ^*∗*^ 10^9^/L, reference value, 150–450) were normal, as were creatinine (0.89 mg/dL, reference value, 0.67–1.17), electrolytes (potassium: 4.22 mmol/L, reference value, 3.45–4.45), liver, and thyroid function tests. Borrelia serology was negative and kininase II (angiotensin converting enzyme, ACE) level was normal (49 U/L, reference value, 8–52).

An isoprenaline (isoproterenol) infusion was started at 0.5 mcg/min and the patient was admitted to the coronary care unit. The isoprenaline infusion was stopped after one day without recurrence of bradycardia. The patient was observed for 4 days, without any further events. ECG at discharge was normal. Echocardiography at presentation and at discharge was normal. Chest X-ray was unremarkable. CT angiography showed no coronary anomaly or stenosis. Cardiac magnetic resonance imaging showed no structural abnormalities or areas with late gadolinium enhancement. Electrophysiological (EP) study was entirely normal and showed a normal atrioventricular conduction time.

Cannabis-induced atrioventricular block was suspected. The patient was advised to quit using cannabis. No further therapy was initiated. Follow-up 5-day ambulatory ECG monitoring, after cessation of cannabis use, showed normal sinus rhythm without atrioventricular block, apart from physiological Wenckebach phenomenon.

## 3. Discussion

We described a young and otherwise healthy patient who presented with third-degree atrioventricular block after using cannabis. The temporal association with exposure and spontaneous resolution after cessation of cannabis use were suggestive for a causal role. Cannabis-induced atrioventricular block remains an elimination diagnosis. Other notable causes, such as hyperkalemia, hypothyroidism, Lyme's disease, sarcoidosis, myocarditis, coronary artery disease, and congenital heart disease, were all excluded in the present case.

Cannabis has become increasingly popular, especially among youngsters. According to the European Monitoring Centre for Drugs and Drug Addiction, 6.8 % of 15- to 64-year olds have used cannabis in the past year and 3.6% in the last month [7]. Decriminalization of cannabis possession and communication about the potential therapeutic properties of medicinal cannabinoids have contributed to the perception of cannabis as a harmless (or even “healthy”) substance. Yet, cannabis is increasingly recognized as a potential underlying cause of cardiovascular emergencies in young patients without other cardiovascular risk factors, including arrhythmia [9–11], myocardial infarction [12], cardiomyopathy, stroke [13], and cardiac arrest [14–17].

The cardiovascular effects of cannabis depend on several factors, including exact composition of the substance, route of administration, dose, and duration of use [18]. Δ^9^‑Tetrahydrocannabinol (THC), the main psychoactive component of cannabis, has biphasic effects on the cardiovascular system [19]. At low to moderate doses, it leads to a surge in sympathetic activity causing tachycardia and hypertension, while parasympathetic activity is predominant at higher doses, causing bradycardia and hypotension.

While cannabis-induced sinus bradycardia is a well-documented phenomenon [6], severe bradycardia leading to (pre)syncope is rare. To date, 10 reports have been published (PubMed search using the strategy: *(“cannabis” OR “marijuana”) AND (“atrioventricular block” OR “av block” OR “heart block” OR “cardiac arrest” OR “bradycardia” OR “asystole” OR “sick sinus syndrome”)*, see Table 1). The bradycardia was sinus arrest in six [20–25], second-degree AV block in two [2, 4], and third-degree AV block in two [3, 5]. With the exception of Menahem et al.'s case [21], all occurred in patients without any past cardiological history. None of the patients were taking any medication. Outcome was favorable in all these reports, with spontaneous resolution after cessation of cannabis exposure. However, cannabis has been implicated as a cause of sudden cardiac death [12, 14, 15, 21, 26]: although most of these deaths are probably linked to myocardial infarction, cannabis-induced bradycardia might have played a role in some.

In conclusion, we reported a case of third-degree atrioventricular block in a young and otherwise healthy patient following cannabis use. Emergency physicians should be aware of the potential cardiovascular side effects of this drug.

## Figures and Tables

**Table 1:** Published cases of documented bradycardia linked to cannabis use.

First author	Year	Title	Gender	Age	Past medical history	Diagnosis
Akins [2]	1981	Marijuana and second-degree AV block	M	21	None	Second-degree AV block
Heeralall [3]	2006	Chronic use presenting as complete heart block	F	46	None	Third-degree AV block
Dockery [20]	2007	Exercise-induced asystole with syncope in a healthy young man	M	40	None	Sinus arrest
Menahem [21]	2013	Cardiac asystole following cannabis (marijuana) usage–additional mechanism for sudden death?	M	21	Total anomalous pulmonary venous drainage, corrected	Sinus arrest
Brancheau [22]	2016	Cannabis induced asystole	M	28	None	Sinus arrest
Robinson [4]	2017	ECG findings in pediatric patients under the influence of marijuana	M	14	None	Second-degree AV block
Grieve-Eglin [23]	2018	Symptomatic sinus arrest induced by acute marijuana use	F	54	CVA	Sinus arrest
Mithawala [5]	2019	Complete heart block from chronic marijuana use	F	51	None	Third-degree AV block
Chaphekar [24]	2019	With a high, comes a low: a case of havy marijuana use and bradycardia in an adolescent	F	17	Anorexia	Sinus arrest
Iqbal [25]	2019	Marijuana induced sick sinus syndrome: a case report	M	27	None	Sinus arrest
Current report	2019	Cannabis-induced complete heart block	M	19	None	Third-degree AV block

## References

[1] Barra S. N., Providencia R., Paiva L., Nascimento J., Marques A. L. (2012). A review on advanced atrioventricular block in young or middle-aged adults. *Pacing and Clinical Electrophysiology*.

[2] Akins D., Awdeh M. R. (1981). Marijuana and second-degree AV block. *Southern Medical Journal*.

[3] Heeralall R., Koradia N., Hoo J., Niranjan S. (2006). 52 Chronic marijuana use presenting as complete heart block. *Journal of Investigative Medicine*.

[4] Robinson J. A., Somasegar S., Shivapour J. K., Snyder C. S. (2018). ECG findings in pediatric patients under the influence of marijuana. *Cannabis*.

[5] Mithawala P., Shah P., Koomson E. (2019). Complete heart block from chronic marijuana use. *The American Journal of Medical Sciences*.

[6] Heckle M. R., Nayyar M., Sinclair S. E., Weber K. T. (2018). Cannabinoids and symptomatic bradycardia. *The American Journal of Medical Sciences*.

[7] Thanki D., Matias J., Griffiths P. (2012). *Prevalence of Daily Cannabis Use in the European Union and Norway*.

[8] Shen J. J., Shan G., Kim P. C., Yoo J. W., Dodge-Francis C., Lee Y.-J. (2019). Trends and related factors of cannabis-associated emergency department visits in the United States: 2006–2014. *Journal of Addiction Medicine*.

[9] Korantzopoulos P. (2014). Marijuana smoking is associated with atrial fibrillation. *The American Journal of Cardiology*.

[10] Khiabani H. Z., Morland J., Bramness J. G. (2008). Frequency and irregularity of heart rate in drivers suspected of driving under the influence of cannabis. *European Journal of Internal Medicine*.

[11] Desai R., Patel U., Deshmukh A., Sachdeva R., Kumar G. (2018). Burden of arrhythmia in recreational marijuana users. *International Journal of Cardiology*.

[12] DeFilippis E. M., Singh A., Divakaran S. (2018). Cocaine and marijuana use among young adults presenting with myocardial infarction: the partners YOUNG-MI Registry. *Journal of American College of Cardiology*.

[13] Singh N. N., Pan Y., Muengtaweeponsa S., Geller T. J., Cruz-Flores S. (2012). Cannabis-related stroke: case series and review of literature. *Journal of Stroke Cerebrovascular Diseases*.

[14] Yankey B. A., Rothenberg R., Strasser S., Ramsey-White K., Okosun I. S. (2017). Effect of marijuana use on cardiovascular and cerebrovascular mortality: a study using the national health and nutrition examination survey linked mortality file. *European Journal of Preventive Cardiology*.

[15] Bachs L., Morland H. (2001). Acute cardiovascular fatalities following cannabis use. *Forensic Science International*.

[16] Davis C., Boddington D. (2015). Teenage cardiac arrest following abuse of synthetic cannabis. *Heart, Lung and Circulation*.

[17] Dines A. M., Wood D. M., Galicia M. (2015). Presentations to the emergency department following cannabis use–a multi-centre case series from ten European countries. *Journal of Medical Toxicology*.

[18] Pacher P., Steffens S., Hasko G., Schindler T. H., Kunos G. (2018). Cardiovascular effects of marijuana and synthetic cannabinoids: the good, the bad, and the ugly. *Nature Reviews Cardiology*.

[19] Menahem S., Preedy V. R. (2017). Cardiovascular effects of cannabis usage. *Handbook of Cannabis and Related Pathologies*.

[20] Dockery B. K., Newman K. P. (2007). Exercise-induced asystole with syncope in a healthy young man. *The American journal of the Medical Sciences*.

[21] Menahem S. (2013). Cardiac asystole following cannabis (marijuana) usage–additional mechanism for sudden death?. *Forensic Science International*.

[22] Brancheau D., Blanco J., Gholkar G., Patel B., Machado C. (2016). Cannabis induced asystole. *Journal of Electrocardiology*.

[23] Grieve-Eglin L., Haseeb S., Wamboldt R., Baranchuk A. (2018). Symptomatic sinus arrest induced by acute marijuana use. *Journal of Thoracic Disease*.

[24] Chaphekar A., Campbell M., Middleman A. B. (2019). With a high, comes a low: a case of heavy marijuana use and bradycardia in an adolescent. *Clinical Pediatrics*.

[25] Iqbal A. M., Mubarik A., Cheetirala V. G., Mohammed S. K., Muddassir S. (2019). Marijuana induced sick sinus syndrome: a case report. *American Journal of Case Reports*.

[26] Hartung B., Kauferstein S., Ritz-Timme S., Daldrup T. (2014). Sudden unexpected death under acute influence of cannabis. *Forensic Science International*.

